# Mechanical ventilation in intensive and critical care units of Russia: RuVent national epidemiologic study

**DOI:** 10.1186/cc10721

**Published:** 2012-03-20

**Authors:** DN Protsenko, AI Yaroshetskiy, SG Suvorov, AU Lekmanov, BR Gelfand

**Affiliations:** 1 Russian National Research Medical University, Moscow, Russia; 2 Moscow Research Institute of Pediatrics and Child Surgery, Moscow, Russia

## Introduction

Experimental data have shown that mechanical ventilation can amplify or possibly trigger lung injury [1,2]. The biggest up-to-date clinical trial by the ARDS Network demonstrates reduction of mortality in ARDS patients with a protective lung strategy [3]. But we can see some gaps between international recommendations and real clinical practice [4,5].

## Methods

The multicenter clinical trial included 470 patients from 101 centers (ICUs) in Russia. Inclusion criteria were all patients without age restrictions ventilated for more than 12 hours for any reason from 14 to 18 February 2011. Recruitment of centers and data collection were made online.

## Results

Total mortality was 35.1%, mortality in ARDS was 44.9%. Prevalence of ARDS was 18.7%. Leading causes for initiation of respiratory support were pathology of the central nervous system (severe TBI 13.3%, stroke 15.7%, craniocephal tumors 5%), sepsis (8.3%), community-acquired pneumonia (8.8%) and ARDS (10.5%). Controlled modes of mechanical ventilation were predominant in our study (A/C 20.2%, SIMV 45.1%, BIPAP 12.6%), other modes includes pressure support ventilation, ASB and PAV. Prevalence of noninvasive respiratory support was only 1.1%. Mean tidal volume calculated by ideal body weight was 8.13 (6.84 to 9.35) for boys and men and 9.1 (7.6 to 10.9) for girls and women. Mean PEEP was 5 (4 to 8) in the whole study and 6 (5 to 9) for ARDS patients.

## Conclusion

Results of the RuVent study are comparable with international epidemiologic multicenter studies. Further investigations are needed for evaluation of the situation in ICUs which are a long distance from big medical centers.
